# Assessing non-invasive glymphatic MRI markers following short-term sleep loss

**DOI:** 10.21203/rs.3.rs-11379946/v1

**Published:** 2026-10-16

**Authors:** Masoud Tahmasian, Jorik Elberse, Daryna Apter, Daniel Aeschbach, Torbjörn Åkerstedt, Andreas Bauer, Florence Borgetto, Kristoffer Brendstrup-Brix, Congying Chu, Nathan Cross, Rachel Custer, Alexander Drzezga, Shohreh Ghorbani, Christophe Grova, Felix Hoffstaedter, Sebastian C Holst, Neda Jahanshad, Hosung Kim, Gitte Knudsen, Sara Ulv Larsen, Abdalla Mohamed, Ju Lynn Ong, Philippe Peigneux, Hanne Smevik, Kai Spiegelhalder, Sandra Tamm, Sophia Thomopoulos, Paul Thompson, Laura Waite, Nathalia Zak, Michael Wei Liang Chee, Jeiran Choupan, Thahn Dang-Vu, David Elmenhorst, Eva-Maria Elmenhorst, Torbjørn Elvsåshagen, Charlotte von Gall, Hans-Peter Landolt, Maiken Nedergaard, Gustav Nilsonne, Alexander Olsen, Govinda Poudel, Whitney Stee, Jeffrey Iliff, Simon Eickhoff

## Abstract

The glymphatic system is a sleep-active pathway involved in clearing metabolic waste from the brain. Several non-invasive MRI indices have been proposed as proxies for assessing glymphatic function *in vivo* . However, it remains unclear whether these indices capture glymphatic dysregulation under short-term sleep loss. Using a large ENIGMA-sleep intervention dataset (n = 429) spanning ten international sites, we examined choroid plexus volume (ChPV), perivascular space volume (PVSV), the free water index (FWI), and the DTI-ALPS index, following habitual sleep (6–8 hours in bed), sleep restriction (3–6 hours in bed), and sleep deprivation (22–40 hours wakefulness). Sleep deprivation was primarily associated with decreased PVSV and ALPS and increased FWI; sleep restriction with the inverse pattern. However, coupling between MRI indices was limited, and models assuming a shared underlying glymphatic system underperformed. Age-related alterations were dissociable from sleep-related alterations and mimicked patterns previously interpreted as glymphatic dysfunction in the literature. Moreover, PVSV increased during the day, whereas ALPS and FWI followed a divergent, anticorrelated trajectory. Our findings suggest that these MRI indices reflect distinct physiological processes; and that sleep loss may influence brain fluid dynamics primarily by modulating inflow resistance through expansion and contraction of the PVS.

m.tahmasian@fz-juelich.de

Research Center Jülich https://orcid.org/0000-0003-3999-3807

Research Centre Juelich

**Daryna Apter**

Research Center Jülich

German Aerospace Center, Institute of Aerospace Medicine

Karolinska Institute https://orcid.org/0000-0002-0117-3793

Concordia University

Neurobiology Research Unit, Copenhagen University Hospital, Rigshospitalet https://orcid.org/0009-0006-8683-4277

**Congying Chu**

National Laboratory of Pattern Recognition, Institute of Automation, Chinese Academy of Sciences https://orcid.org/0000-0002-7808-8896

**Nathan Cross**

The University of Sydney https://orcid.org/0000-0002-7325-7646

University of Southern California

University Hospital Cologne https://orcid.org/0000-0001-6018-716X

National University of Singapore

Physics dpt. and PERFORM Centre, Concordia Univ.; Biomedical Eng. dpt., McGill Univ.

Research Centre Juelich https://orcid.org/0000-0001-7163-3110

**Sebastian C Holst**
Roche (Switzerland)

**Neda Jahanshad**
Imaging Genetics Center, Mark and Mary Stevens Neuroimaging and Informatics Institute, Keck School of Medicine of USC, Marina del Rey, CA https://orcid.org/0000-0003-4401-8950

**Hosung Kim**
University of Southern California https://orcid.org/0000-0002-2269-8644

**Gitte Knudsen**
Copenhagen University Hospital, Rigshospitalet

**Sara Ulv Larsen**
Copenhagen University Hospital, Rigshospitalet https://orcid.org/0000-0002-9046-2430

**Abdalla Mohamed**
University of the Sunshine Coast

**Ju Lynn Ong**
National University of Singapore

**Philippe Peigneux**
Universite Libre de Bruxelles (ULB) https://orcid.org/0000-0003-4745-1434

**Hanne Smevik**
Norwegian University of Science and Technology

**Kai Spiegelhalder**
University Hospital of Freiburg

**Sandra Tamm**
Karolinska Institutet

**Sophia Thomopoulos**
University of Southern California https://orcid.org/0000-0002-0046-4070

**Paul Thompson**
Imaging Genetics Center, Mark and Mary Stevens Neuroimaging & Informatics Institute, Keck School of Medicine of the University of Southern California, Marina del Rey, California, USA
https://orcid.org/0000-0002-4720-8867

**Laura Waite**
Research Centre Jülich

**Nathalia Zak**
Oslo University Hospital

**Michael Wei Liang Chee**
National University of Singapore https://orcid.org/0000-0002-6087-0548

**Jeiran Choupan**
University of Southern California https://orcid.org/0000-0002-4760-7796

**Thahn Dang-Vu**

Concordia University https://orcid.org/0000-0002-7235-2721
**David Elmenhorst**
Institute of Neuroscience and Medicine, Molecular Organization of the Brain (INM-2), Forschungszentrum Jülich, Jülich https://orcid.org/0000-0001-6137-416X
**Eva-Maria Elmenhorst**
German Aerospace Center, Institute of Aerospace Medicine https://orcid.org/0000-0003-0336-6705
**Torbjørn Elvsåshagen**
Oslo University Hospital https://orcid.org/0000-0001-9991-1610
**Charlotte von Gall**
Heinrich Heine University Düsseldorf https://orcid.org/0000-0002-5088-3118
**Hans-Peter Landolt**
University of Zurich, Switzerland https://orcid.org/0000-0003-0887-9403
**Maiken Nedergaard**
University of Copenhagen https://orcid.org/0000-0001-6502-6031
**Gustav Nilsonne**
Karolinska Institutet https://orcid.org/0000-0001-5273-0150
**Alexander Olsen**
Norwegian University of Science and Technology
**Govinda Poudel**
Australian Catholic University https://orcid.org/0000-0002-0043-7531
**Whitney Stee**
Université Libre de Bruxelles
**Jeffrey Iliff**
University of Washington School of Medicine https://orcid.org/0000-0002-6910-0080
**Simon Eickhoff**
Research Centre Jülich

**Article**

**Posted Date:** October 16th, 2026

**License:** This work is licensed under a Creative Commons Attribution 4.0 International License.
Read Full License

**Additional Declarations: Yes** there is potential Competing Interest. The authors declare no competing interests directly related to this work. S.C.H. is a full time employee of Hoffmann-La Roche. A.O. is a cofounder and share holder of Nordic Brain Tech AS.

## Assessing non-invasive glymphatic MRI markers following short-term sleep loss

Jorik Daniël Elberse[1,2,3], Daryna Apter[1,4], Daniel Aeschbach[5], Torbjörn Åkerstedt[6,7], Andreas Bauer[8], Florence Borgetto[9], Kristoffer Brendstrup-Brix[10,11], Congying Chu[8,12], Nathan Cross[9], Rachel Custer[13], Alexander Drzezga[8,14], Shohreh Ghorbani[15], Christophe Grova[9], Felix Hoffstaedter[1,2], Sebastian C. Holst[16], Neda Jahanshad[13], Hosung Kim[17], Gitte M. Knudsen[10,11], Sara M. Ulv Larsen[10,18], Abdalla Z. Mohamed[19], Ju Lynn Ong[15], Philippe Peigneux[20,21], Hanne Smevik[22,23], Kai Spiegelhalder[24], Sandra Tamm[6], Sophia I. Thomopoulos[25], Paul M. Thompson[25], Laura Waite[1,2,], Nathalia Zak[26,27], Michael W.L. Chee[15], Jeiran Choupan[13], Thien Thanh Dang-Vu[9,28,29,30], David Elmenhorst[8,14], Eva-Maria Elmenhorst[5,31], Torbjørn Elvsåshagen[26,27,32], Charlotte von Gall[33], Hans-Peter Landolt[34,35], Maiken Nedergaard[36,37], Gustav Nilsonne[6,7], Alexander Olsen[22,23,37], Govinda Poudel[38], Whitney Stee[20,21], Jeffrey J. Iliff[39,40,41], Simon B. Eickhoff[1,2], and Masoud Tahmasian[1,2,14]*, for the ENIGMA-Sleep Working Group.

1. Institute of Neuroscience and Medicine, Brain and Behavior (INM-7), Research Center Jülich, Jülich, Germany.
2. Institute of Systems Neuroscience, Medical Faculty and University Hospital Düsseldorf, Heinrich Heine University, Düsseldorf, Germany.
3. Max Planck School of Cognition, Max Planck Institute for Human Cognitive and Brain Sciences, Leipzig, Germany.
4. Department of Child and Adolescent Psychiatry, Psychosomatics and Psychotherapy, Faculty of Medicine and University Hospital Cologne, University of Cologne, Cologne, Germany.
5. Institute of Aerospace Medicine, German Aerospace Center, Cologne, Germany.
6. Department of Clinical Neuroscience, Karolinska Institutet, Stockholm, Sweden.
7. Department of Psychology, Stockholm University, Stockholm, Sweden.
8. Institute of Neuroscience and Medicine, Molecular Organization of the Brain (INM-2), Research Center Jülich, Jülich, Germany.
9. Sleep, Cognition, and Neuroimaging Laboratory (SCNLab), Department of Health, Kinesiology, and Applied Physiology, Concordia University, Montreal, Canada.
10. Neurobiology Research Unit, Copenhagen University Hospital Rigshospitalet, Copenhagen, Denmark.
11. Department of Clinical Medicine, Faculty of Health and Medicinal Sciences, University of Copenhagen, Denmark.
12. National Key Laboratory of Cognitive Neuroscience and Learning; IDG/McGovern Institute for Brain Research, Beijing Normal University, Beijing, China.
13. Mark and Mary Stevens Neuroimaging and Informatics Institute, Keck School of Medicine, University of Southern California, CA, USA.
14. Department of Nuclear Medicine, Faculty of Medicine and University Hospital Cologne, University of Cologne, Cologne, Germany
15. Centre for Sleep and Cognition, Yong Loo Lin School of Medicine, National University of Singapore, Singapore.
16. Roche Pharma Research and Early Development, Neuroscience and Rare Diseases, Roche Innovation Center Basel, F. Hoffmann–La Roche Ltd., Basel, Switzerland.
17. Department of Neurology, USC Stevens Neuroimaging and Informatics Institute, Keck School of Medicine, University of Southern California, Los Angeles, California, USA.
18. Department of Psychiatry and Behavioral Sciences, University of Washington School of Medicine, Seattle, WA, USA.
19. Department of Cognitive Sciences, College of Humanities and Social Sciences, United Arab Emirates, Al Ain, Abu Dhabi, United Arab Emirates.

20. UR2NF-Neuropsychology and Functional Neuroimaging Research Unit affiliated at CRCN – Centre for Research in Cognition and Neurosciences and UNI - ULB Neuroscience Institute, Université Libre de Bruxelles, Brussels, Belgium.
21. GIGA - Cyclotron Research Centre - In Vivo Imaging, University of Liège (ULiège), Liège, Belgium.
22. Department of Psychology, Norwegian University of Science and Technology, Trondheim, Norway.
23. Norwegian Centre for Headache Research (NorHead), Norwegian University of Science and Technology, Trondheim, Norway.
24. Department of Psychiatry and Psychotherapy, Medical Center Freiburg, Germany.
25. Imaging Genetics Center, Mark and Mary Stevens Neuroimaging and Informatics Institute, Keck School of Medicine, University of Southern California, CA, USA.
26. Center for Precision Psychiatry, Institute of Clinical Medicine, University of Oslo Department of Neurology, Oslo University Hospital, Oslo, Norway.
27. Department of Behavioural Medicine, Institute of Basic Medical Sciences, University of Oslo, Norway.
28. Centre for Studies in Behavioral Neurobiology, Concordia University, Montreal, Canada.
29. Centre de Recherche de l'Institut Universitaire de Gériatrie de Montréal (CRIUGM), CIUSSS Centre-Sud-de-l'Ile-de-Montréal, Montréal, Canada.
30. School of Health, Concordia University, Montreal, Canada.
31. Institute of Occupational, Social, and Environmental Medicine, RWTH Aachen University, Aachen, Germany.
32. Department of Neurology, Division of Clinical Neuroscience, Oslo University Hospital, Oslo, Norway.
33. Institute of Anatomy II, Medical Faculty, Heinrich Heine University, Düsseldorf, Germany.
34. Institute of Pharmacology and Toxicology, Human Sleep Psychopharmacology Laboratory, University of Zurich, Switzerland.
35. Sleep and Health Zurich, University of Zurich, Zurich, Switzerland.
36. Center for Translational Neuromedicine, University of Copenhagen, Copenhagen, Denmark.
37. Clinic of Rehabilitation, St. Olavs Hospital, Trondheim University Hospital, Trondheim, Norway.
38. Mary Mackillop Institute for Health Research, Faculty of Health Sciences, Australian Catholic University, Melbourne, Australia.
39. Department of Neurology, University of Washington School of Medicine, Seattle, WA, USA.
40. VISN 20 Northwest Mental Illness Research, Education and Clinical Center, VA Puget Sound Healthcare System, Seattle, WA, USA.
41. Department of Psychiatry and Behavioral Sciences, University of Washington School of Medicine, Seattle, WA, USA.

***Corresponding Author:** Institute of Neuroscience and Medicine, Brain & Behaviour (INM-7), Research Centre Jülich, Wilhelm-Johnen-Straße, 52428 Jülich, Germany. Phone: +49 2461 61-8785, Fax: +49 2461 61-1880. Email: m.tahmasian@fz-juelich.de

**Keywords:** Sleep deprivation, Sleep restriction, Glymphatic system, Perivascular Space, Choroid Plexus, Free Water, DTI-ALPS

## Abstract

The glymphatic system is a sleep-active pathway involved in clearing metabolic waste from the brain. Several non-invasive MRI indices have been proposed as proxies for assessing glymphatic function *in vivo*. However, it remains unclear whether these indices capture glymphatic dysregulation under short-term sleep loss. Using a large ENIGMA-sleep intervention dataset (n=429) spanning ten international sites, we examined choroid plexus volume (ChPV), perivascular space volume (PVSV), the free water index (FWI), and the DTI-ALPS index, following habitual sleep (6-8 hours in bed), sleep restriction (3-6 hours in bed), and sleep deprivation (22-40 hours wakefulness). Sleep deprivation was primarily associated with decreased PVSV and ALPS and increased FWI; sleep restriction with the inverse pattern. However, coupling between MRI indices was limited, and models assuming a shared underlying glymphatic system underperformed. Age-related alterations were dissociable from sleep-related alterations and mimicked patterns previously interpreted as glymphatic dysfunction in the literature. Moreover, PVSV increased during the day, whereas ALPS and FWI followed a divergent, anticorrelated trajectory. Our findings suggest that these MRI indices reflect distinct physiological processes; and that sleep loss may influence brain fluid dynamics primarily by modulating inflow resistance through expansion and contraction of the PVS.

## Introduction

The glymphatic system stands out as one of the most influential and widely debated models in modern sleep research [1,2]. Much of its prominence stems from its explanatory potential, as it provides a framework for understanding protective effects of non-rapid eye movement (NREM) sleep in the context of aging and various neuropsychiatric disorders [3]. The glymphatic model comprises five sequential steps: cerebrospinal fluid (CSF) produced by choroid plexus (ChP) tissue flows from the ventricles into the subarachnoid space surrounding the cortex and is then driven into the perivascular spaces (PVS) of penetrating arteries by arterial pulsation, respiration, and slow vasomotion. Aquaporin-4 (AQP4) water channels, located on perivascular astrocytic endfeet, mediate the transfer of CSF-derived fluid into the brain parenchyma. Within the parenchyma, this fluid exchanges with interstitial fluid (ISF) through diffusion and convective flow, facilitating nutrient delivery and metabolic waste clearance. Finally, this mixture of ISF and metabolites is taken up by the PVS of large-caliber draining veins and directed towards the dural venous sinuses. From there, waste is transported out of the brain via the meningeal lymphatic vessels to the cervical lymph nodes [4,5]. Studies in rodents and humans have demonstrated that proteins such as amyloid-beta, tau, and alpha-synuclein may be cleared along glymphatic pathways [4,6,7]. Consistent with this, disruption of AQP4 function is sufficient to promote protein mis-aggregation in transgenic mouse models [8,9], while changes in its expression and localization are associated with pathological burden in *post-mortem* human tissues [10]. Dysregulation of glymphatic function has been implicated in a broad set of pathologies, including neurodegeneration, traumatic brain injury, hydrocephalus, stroke, and epilepsy [11].

The glymphatic system is likely sleep-active [5,6,12,13]. In murine models, glymphatic activity increases markedly under natural sleep conditions and delta-wave-dominant anesthesia [12]. Extracellular spaces expand during sleep, reducing resistance to brain fluid flow, while they contract during wakefulness [12,14]. Infraslow oscillations in norepinephrine (NE) tone promote glymphatic activity by modulating slow vasomotion of cerebral blood vessels, which drives CSF flow through PVSs during sleep [12,15]. Glymphatic exchange of CSF and ISF, as well as AQP4 polarization, are under circadian regulation [16,17]. Additionally, stimulation of low-frequency neural oscillations like those present during NREM sleep enhances glymphatic clearance in mice [18]. In humans, solute clearance from the interstitium is significantly impaired after short-term sleep loss [6,13], with solutes remaining in the brain for up to 48 hours following total deprivation [13].

Much of the human glymphatic literature relies on invasive studies with relatively small sample sizes. To overcome this, non- or minimally invasive proxies have been proposed to assess glymphatic function in larger clinical studies [19]. Anatomical MRI sequences such as T1-weighted (T1w), T2-weighted (T2w), and/or fluid-attenuated inversion recovery (FLAIR) can be used to segment MRI-visible PVS, whose enlargement is a possible marker of (advanced) aging and may correlate with alterations in CSF influx and AQP4 polarization [20,21]. These structural sequences can also be used to obtain a ChP segmentation, whose enlargement is proposed to reflect changes in brain-wide CSF dynamics [22]. Diffusion MRI (dMRI) enables quantification of free water (FW) content within the brain parenchyma and estimation of diffusion along perivenous spaces, as assessed by DTI- and DKI-ALPS indices [19,23,24]. While these proxy approaches are designed to mitigate prior methodological caveats, they present certain trade-offs. For instance, many fail to directly capture glymphatic solute exchange, thereby raising questions regarding validity [25]. In addition, their sensitivity to changes in brain morphology obscures whether the observed effects reflect glymphatic dysfunction or age-related atrophy of white or gray matter, an issue of particular relevance in geriatric and neurodegenerative samples [25,26]. Nevertheless, these methods have yielded valuable insights in the past decade, with several studies characterizing glymphatic (dys)regulation based on the results of one or a few of these proxies [27–29].

Given that sleep is a significant modulator of the glymphatic system, this pre-registered study (https://osf.io/w4kx3) aims to investigate the effect of experimental sleep loss on four widely used glymphatic MRI indices: the choroid plexus volume (ChPV), the perivascular space volume (PVSV), the free-water index (FWI), and the DTI-ALPS index. The present study combines datasets from ten international sites within the ENIGMA-Sleep Working Group, each implementing either a sleep restriction (SR; i.e., delayed sleep onset) or sleep deprivation (SD; i.e., prolonged forced wakefulness) protocol. We hypothesized that i) greater sleep loss would correlate with increased ChPV, PVSV, FWI, and reduced ALPS, a pattern previously considered indicative of glymphatic dysfunction [19]; ii) integrating these indices into a single composite index would better capture system-wide alterations across the glymphatic system and outperform conventional single-feature models; iii) age and time of day would act as significant covariates within these models.

## Results

### Participant Demographics

An overview of the study protocol is presented in **Figure 1.** A total of $n$ = 429 participants was collected from 10 international sites (**Table 1**), including 209 female and 220 male participants, with a mean age of 30.1 ± 14.5 years. Of these participants, 38 were normal sleep (NS) controls who had completed two nights of habitual sleep. The remaining participants belonged to either the SR or SD groups. The SR group (n = 205) completed one night of habitual sleep followed by either acute (one night) or chronic (five nights) reduction of time spent in bed, whereas the SD group (n = 186) completed one night of habitual sleep followed by one night of continuous wakefulness. Average sleep duration for the follow-up visit was estimated at around 7.0 ± 1.0 hours in the control group and 4.0 ± 1.0 hours in the SR group. Participants in the SD group were deprived of sleep for an estimated 28.3 ± 6.8 hours.

The sociodemographic and clinical differences between all experimental groups are displayed in **Table 2**. There were significant differences in age, sex, chronotype, and time of MRI acquisition between all groups (all $p$s < .05). On average, SR participants were the oldest (34.2 ± 17.6 years) and SD participants the youngest (26.0 ± 10.0 years). Women were more prevalent in the control group (66%), and men in the SD group (58%). Additionally, morning-types were more prevalent than evening-types across all groups (24% vs. 16%). Intermediate chronotypes were most prevalent overall (60%). Finally, the mean acquisition time (calculated over time of day in seconds) in the SR group was significantly later (14:13 ± 4 hours 30 minutes) than the control (10:44 ± 1 hour 20 minutes) and SD groups (10:55 ± 2 hours 47 minutes).

**Sleep Loss and MRI Index Alterations**

Subjective sleepiness, as measured by the Karolinska Sleepiness Score (KSS), increased following acute/chronic SR (henceforth SR; ß = 4.90, SE = .19, $z$ = 26.14, $p$ < .001) and SD (ß = 6.66, SE = .22, $z$ = 30.89, $p$ < .001), as expected (**Figure S1**). We assessed concurrent alterations across four MRI indices, ChPV, PVSV, FWI, and DTI-ALPS (henceforth ALPS), by normalizing the indices and sign-inverting ALPS, to obtain a composite index where higher values reflect greater abnormality according to our *a priori* hypotheses. This composite index increased following SD (ß = .60, SE = .11, $t$ = 5.00, $p$ < .001) and decreased following SR (ß = -.38, SE = .10, $t$ = -3.95, $p$ = .001, **Figure 2b**). The main effect of SD was resilient to site-based variability, as it was maintained across 9 iterations of leave-one-site-out (LOSO) bootstrapping (highest $p$ = .004). The significant effect of SR was maintained across 8 iterations, with the exclusion of the Juelich site (n = 39) yielding a non-significant result. Neither models could converge when the Trondheim site was excluded due to insufficient contrasts. Interestingly, the composite index model underperformed across nearly all evaluation metrics (AIC = 5148.8, BIC = 5246.4, $R^2_{cond}$ = .377, $R^2_{marg}$ = .223 RMSE = .633) compared to modeling MRI indices individually (**Figure 2a, Table S1**). The composite model demonstrated superior performance only in terms of the marginal $R^2$, reflecting a greater proportion of variance explained by predominantly between-subject fixed effects, including sleep condition and age.

We evaluated the within- and between-subject differences in MRI index alterations for each sleep loss condition using the estimated marginal means (EMMs). Within the composite model, EMMs showed differences between the NS, SR, and SD conditions for PVSV, FWI, and ALPS, but not ChPV (**Figure 2c**). The EMMs of PVSV were negative at baseline (EMM = -.20, 95% CI [-.36, -.04]), with further reduction after SD (EMM = -.25, 95% CI [-.43, -.08]), and near-zero values in SR (EMM = .00, 95% CI [-.18, .19]). Pairwise comparisons revealed an increase in PVSV from NS to SR ($\Delta$ = .20, $d$ = .29, $p$ = .003) and a reduction when comparing SR to SD ($\Delta$ = -.26, $d$ = -.43, $p$ = .003). The contrast between NS and SD approached significance after adjustment ($p$ = .062). The SR group exhibited a decreased FWI compared to NS (EMM = -.58, 95% CI [-.81, -.35]; $\Delta$ = -.68, $d$ = -1.1, $p$ < .001), while the SD group showed no significant alterations. The contrast between SD and SR was also significant ($\Delta$ = -.68, $d$ = -.91, $p$ < .001). The (sign-corrected) ALPS index was increased in the SR group relative to baseline (EMM = .39, 95% CI [.16, .62]; Δ = .26, $d$ = .47, $p$ = .008) and decreased in the SD group (EMM = -.42, 95% CI [-.67, -.19]; Δ = -.55, $d$ = -.75, $p$ < .001). The difference between SD and SR was again significant (Δ = -.82, $d$ = -1.2, $p$ < .001). Single-index models reproduced the reductions in PVSV and ALPS in the SD condition and the increase in FWI in the SR condition (**Figure S3**).

**Age and MRI Index Alterations**

The composite index was positively associated with age (ß = 0.44, SE = .001, $t$ = 19.7, $p$ < .001, **Figure 2b**) in accordance with our hypotheses. Analyses of individual indices further showed that ChPV, PVSV, and FWI increased with age, whereas ALPS did not reveal an age-related change (**Figure 3, Figure S3**). Crucially, the diffusion indices included no participants older than 45, limiting the generalizability of this index. In contrast, the anatomical indices included participants aged 65–70; individuals older than 70 had been top-coded as “70+” in prior analyses. A post hoc model incorporating the brain parenchymal fraction (BPF; i.e. the ratio of GM and WM volume to ICV) as an interaction term with age yielded no significant moderating effect (p = .369), indicating that brain morphology does not fully account for this observed relationship between age and MRI index alterations (**Table S1c**).

**Time of Day and MRI Index Alterations**

Contrary to our hypotheses, the association between the composite index and MRI acquisition time was non-significant (**Figure 2b**). Nevertheless, analyses of individual indices indicated that PVSV and ALPS tended to increase with later acquisition times (ß = .09, SE = .02, $t$ = 3.97, $p$ < .001; ß = .15, SE = .05, $t$ = 3.23, $p$ = .005; **Figure S3**). The lack of a significant effect in the main model may reflect asynchrony in the diurnal fluctuations of these indices, reducing their reliability for assessment using linear models. To investigate this further, we leveraged the spread of acquisition times (**Figure S5**) and aggregated MRI index values into 30-minute bins, using one-sample t-tests in each bin to assess whether the mean values differed from zero. We chose 30 minutes because it provided the best trade-off between temporal resolution and data saturation across bins. Pseudo-time course plots were created separately for the NS, SR, and SD conditions to avoid confounding by sleep quality of the prior night (**Figure 4, Figure S6**). Following NS, ChPV exhibited little fluctuation, with modest reductions in early morning bins and an isolated significant increase in the evening (**Figure 4a**). In contrast, PVSV was significantly reduced across most morning and afternoon time bins, with higher values observed in the evening. Most notably, FWI and ALPS appeared to exhibit opposing temporal trends, with lower FWI and higher ALPS before noon, a reversal around midday, and a second reversal in the afternoon. Observations following SD revealed largely comparable patterns: ChPV showed morning reductions but otherwise remained mostly stable; PVSV was reduced in the morning and early afternoon; and FWI and ALPS appeared to follow opposing temporal trends with a transition before noon (**Figure 4b**). Insufficient data precluded assessment of changes in ChPV and PVSV in the evening. Observations following SR were highly irregular, with gaps in intermediate bins arising from the group's wide range of acquisition times (**Figure S5, Table 2**). Nevertheless, there was limited indication of a morning-to-evening increase in PVS and an anticorrelation between FWI and ALPS, albeit without the midday shift observed following NS and SD (**Figure S6**).

### Impact of ChPV on Composite Index Performance

In the sections above, we showed that PVSV, FWI, and ALPS are modulated by sleep, age, and time of day. In contrast, ChPV showed a significant relationship with age alone; time of day was not significant in the main model, and per-bin tests revealed only small deviations from baseline. This suggests that the inclusion of the ChpV may adversely affect the performance of the composite index, which was intended to capture acute fluctuations in the glymphatic system. To verify this, we refitted the main model with a composite index derived solely from PVSV, FWI, and ALPS data. This modification led to marked improvements in model fit ($R^2_{cond}$ = .459, $R^2_{marg}$ = .249, RMSE = .517, **Table S1c**). The effects of SR, SD, and age were maintained (ß = -.25, SE = .08, $t$ = -2.43, $p$ = .018; ß = .67, SE = .09, $t$ = 5.64, $p$ < .001; ß = .40, SE = .002, $t$ = 14.0, $p$ < .001). As expected, the effect of MRI acquisition time became significant (ß = .07, SE = .02, $t$ = 2.31, $p$ = .023), with later acquisition times associated with a higher composite index. However, modeling each index individually remained optimal, which may explain the improved model fit observed after ChpV exclusion.

### Correlations with Clinical and Cognitive Variables

The modest accuracy of the composite model, along with variations across age and time of day, suggests a more complex structure beneath the presented data. To further investigate these patterns, we created a correlogram using the MRI indices and a subset of cognitive and clinical variables selected for their availability across most datasets. Among indices, ChPV was positively correlated with PVSV and ALPS; PVSV was negatively correlated with FWI; and FWI was negatively correlated with ALPS (**Figure 5a**). The correlations between ChPV and FWI, and between PVSV and ALPS, were non-significant. Age and vigilance (PVT-RS) were positively associated with PVSV, while sleepiness (KSS) and sleep quality (PSQI) were negatively associated, indicating divergent age-related and sleep-related effects. FWI was positively associated with age, BMI, and PSQI, but negatively associated with KSS; ALPS was also positively associated with KSS. As expected, KSS was positively correlated with PSQI and negatively with PVT-RS. None of the indices showed significant differences between the sexes (**Figure 5b**). Finally, PVSV correlated significantly with chronotype, with evening-types exhibiting the highest values and intermediate-types the lowest. All post hoc correlations showed consistently low to modest effect sizes (**Figure 5a**).

### Impact of Acute vs. Chronic Sleep Restriction

In a post hoc analysis independent of the main model, acute (one night) SR (aSR) and cumulative (three to five nights) SR (cSR) were compared for the subset of measures with sufficient data. Datasets with three to five nights of SR were pooled to avoid confounding with site (**Table S4**). As reported above, self-reported sleepiness increased monotonically from NS to aSR, cSR, and SD (**Figure S1**). Vigilance increased monotonically from aSR to cSR to SD (**Figure S2**). Although ChPV was stable across NS and SR in the main analyses, the present sub-analyses revealed a significant reduction in ChPV specifically following aSR ($\Delta = -.31$, $d = -.49$, $p < .001$, **Figure S7**). Moreover, the PVSV appeared to be increased following aSR ($\Delta = .24$, $d = .38$, $p = .005$) but decreased following cSR ($\Delta$ = -.24, $d$ = -.38, $p$ = .005). While MRI index alterations following aSR were more consistent with the effects observed in the primary analysis, those following cSR most closely resembled the pattern seen following SD.

## Discussion

The present study evaluated four potential non-invasive MRI indices of glymphatic function in a large sleep restriction and sleep deprivation dataset. It was hypothesized that increasing sleep loss would correlate with increased ChPV, PVSV, and FWI, as well as decreased ALPS, a pattern previously theorized to reflect glymphatic dysfunction [19]. While the concept of "glymphatic dysfunction" remains incompletely defined, it is commonly characterized by reduced efficiency of solute exchange between CSF and ISF, and between the brain parenchyma and blood plasma. To date, these processes have largely been measurable only through invasive methods in humans and animals. Non-invasive proxy measures, therefore, represent an important step towards large-scale clinical assessment of the glymphatic system in humans. An influential study by Kamagata et al. [30] validated these indices in an Alzheimer's disease cohort and proposed a unified framework for assessing glymphatic influx, parenchymal flow, and efflux entirely through non-invasive proxies. They hypothesized that PVS enlargement was a marker of reduced AQP4 function, thus attenuating transport of CSF-derived fluid into the parenchyma. This would reduce the rate of bulk flow and solute exchange within the parenchyma, as reflected by a high FWI, and the rate of diffusion along perivenous efflux channels, as reflected by a low ALPS index. Indeed, they found that individuals with clinical Alzheimer's disease exhibited high PVSV and FWI and low ALPS compared to individuals with mild cognitive impairment and cognitively normal controls, concluding that this was evidence of glymphatic dysfunction. However, our findings challenge this model in several respects: first, separate modeling of MRI indices improved accuracy over a composite approach assuming a shared latent system (**Figure 1a**). Second, coupling between indices was limited and only evident for FWI-ALPS and, to a lesser extent, for ChPV-PVSV (**Figure 5a**). Finally, the effects of acute sleep deprivation, sleep restriction, and age were equally strong and clearly dissociable (**Figure 1b, Figure 2**). Whereas age-related alterations mirrored those reported by Kamagata et al., both sleep-reduction protocols produced distinct changes across MRI indices. Taken together, our findings suggest that these indices capture aspects of brain physiology and structure that may be relevant to solute exchange, yet contradict the notion that they are valid, composite markers of glymphatic function. In the following paragraphs, we discuss the implications of the observed changes in relation to experimental sleep loss, age, and time of day.

Acute alterations in PVSV in response to sleep loss remain poorly characterized in healthy populations. Prior work using the Stockholm dataset demonstrated increased CSO-PVS following one night of sleep restriction in young adults [31]. Several studies have observed that higher sleep efficiency is associated with greater PVSV in cognitively normal older adults [32,33]. Here, we show for the first time that total sleep deprivation was associated with reduced CSO- and BG-PVSV. PVS size and shape influence CSF dynamics and glymphatic exchange by modulating inflow resistance, with reduced PVSV increasing resistance and limiting CSF entry into the system. Moreover, PVS morphology may also affect other mechanisms of fluid-mediated waste clearance, including intramural periarterial drainage (IPAD) [34]. Our findings suggest that a single night of total sleep deprivation may significantly increase resistance to fluid flow within the PVS, potentially impairing the capacity for solute delivery and clearance. This is consistent with prior work showing an overall reduction in extracellular space volume during prolonged wakefulness [6,12]. Sleep loss may also impact breathing, vascular tone, and noradrenergic signalling, which are all key drivers of CSF inflow [13,15]. Rather than representing glymphatic degradation, this acute contraction of PVS may reflect an active, state-dependent vascular adaptation in healthy individuals, driven by noradrenergic arousal to sustain wakefulness against excessively highly sleep propensity as a consequence of total deprivation. This interpretation would be consistent with the PVSV alterations observed following acute and chronic sleep restriction, as partial sleep pressure may expand PVS until a hyper-adrenergic “survival” state is induced. In this state, sleep-active fluid transport would be downregulated by reducing vasomotion and raising inflow resistance. Further studies should investigate the relationship between PVSV, NE signalling, and vasomotion in total sleep deprivation through multimodal protocols including pupillometry, locus coeruleus fMRI, or pharmacological NE manipulation with drugs such as zolpidem [15].

Prior studies have linked total sleep deprivation to changes in WM microstructure and reduced fractional anisotropy [35]. Accordingly, we found that sleep deprivation increased the WM FWI. This increase may reflect a shift towards greater isotropic water content within the WM, potentially driven by altered interstitial fluid homeostasis during prolonged wakefulness. Interestingly, *post hoc* analyses revealed a significant negative coupling between FWI and PVS alterations, possibly supporting the notion that increased inflow resistance leads to impaired exchange between the PVS and parenchyma, though direct measurements of intercompartmental fluid exchange would be required to confirm this. FWI further exhibited a strong negative coupling with the ALPS index. ALPS reductions have previously been observed in individuals with poor self-reported sleep quality [36] and in the context of various sleep disorders, including insomnia [37,38], obstructive sleep apnea [38,39], REM behavior disorder [40], and narcolepsy type 1 [41]. As expected, we identified a reduction in ALPS following sleep deprivation. While this pattern has often been interpreted as reflecting reduced CSF efflux along the deep medullary veins, our data cannot support or contradict this interpretation. We did, however, find that coupling between PVSV and ALPS was non-significant, suggesting that these alterations may be occurring independently of one another. This is in line with recent work by Mossige et al. [42], which found no relationship between ALPS and tracer enrichment in contrast-enhanced MRI. It is possible that both FWI and ALPS capture non-specific, state-dependent fluctuations in brain-wide fluid dynamics, which may underlie their strong association. Similar to the PVSV findings, one night of sleep restriction showed the inverse pattern to sleep deprivation across both FWI and ALPS; however, these results were obtained from a single site and require replication in independent cohorts to ensure reliability.

Each of the MRI indices investigated here are known to vary with age. Enlargement of the ChP in advanced age may be explained by progressive stromal fibrosis, membrane thickening, and calcification [43]. It remains contentious whether this enlargement also represents a compensatory response to reduced perfusion and impaired CSF circulation [22,43]. Enlargement of the PVS is another well-established marker of aging and is frequently observed in preclinical models of disorders characterized by impaired waste clearance, such as small-vessel disease and neurodegenerative disorder. Factors believed to contribute to this enlargement include arterial stiffening, blood-brain barrier leakage, fluid stagnation, and inflammation and atrophy of surrounding tissue [44]. These factors may similarly impact white matter free water content, together with demyelination and altered interstitial fluid exchange [45]. Additionally, the ALPS index strongly correlates with aging due to its sensitivity to changes in fluid dynamics as well as WM volume and fiber integrity [26]. In the present study, age-related changes across the ChP, PVS, and FWI were largely consistent with the existing literature [43–46]. We did not, however, observe a significant relationship between age and the ALPS index. A likely reason for this observation is the relatively limited age range for the diffusion MRI analyses, which included no individuals older than 46 years and consisted predominantly of participants aged 20-30 years. Follow-up studies with broader age distributions are therefore warranted. The present study largely corroborates the concerns regarding non-invasive MRI indices laid out in reviews such as Keil et al. [25], namely that aging and morphological alterations of the brain, not glymphatic dysfunction, may account for a substantial proportion of previously reported observations. Indeed, the pattern of alterations observed in our study neatly maps onto the model proposed by Kamagata et al. [30], and aligns with numerous other studies that reported MRI index alterations in aging [47], neurodegenerative disease [30,48], and various other neuropathologies where age may affect the severity of impairment [29]. However, a post hoc analysis incorporating brain parenchymal fraction as an interaction term with age revealed no significant moderation effect, despite a strong negative correlation between brain parenchymal fraction and age, suggesting that brain morphological alterations do not fully account for the observed relationship.

Diurnal modulation of non-invasive MRI indices remains largely unexplored, despite evidence that glymphatic and related fluid exchange processes are strongly influenced by circadian regulation. Barisano et al. [49] observed that PVS volumes generally rise throughout the day. Similarly, Ko et al. [50] reported time-of-day–dependent fluctuations in the ALPS index, with peak values in the morning, lower values during the day, and a rebound in the evening. In the present study, we examined the associations between MRI indices and diurnal time. In the absence of dense within-subject sampling, we leveraged the large sample size and variability in acquisition times of our data to approximate a temporal profile of MRI indices across the day. Our findings appear to replicate those of Barisano et al. and Ko et al., showing a time-dependent increase in PVSV alongside a significant morning-to-daytime shift in ALPS values following both normal sleep and sleep deprivation. The latter appears to also be accompanied by an anticorrelated shift in the FWI, suggesting that these diurnal changes in diffusion metrics are not restricted to the perivascular compartment of the deep medullary veins. Diurnal fluctuations in the ChP volume were limited. Moreover, we were unable to replicate the above findings in the sleep restriction condition; however, this time course exhibited a much broader distribution of acquisition times, resulting in significantly fewer observations per epoch. Taken together, the present study corroborates the theory that PVSV, FWI, and ALPS demonstrate a degree of diurnal fluctuations. It also offers initial evidence for a temporal coupling between FWI and ALPS. These results all require further validation through dedicated study protocols, including within-subject dense sampling.

Our findings highlight a marked contrast between robust age-related changes and transient, state-dependent fluctuations associated with sleep and diurnal time across MRI indices. It supports their potential as relevant clinical markers, yet also emphasizes the need for rigorous control of sleep quality and time of day to distinguish between acute and chronic alterations. While these indices may not directly measure glymphatic function, they do capture structural brain features that may affect CSF influx and exchange processes; consequently, meaningful interpretation in a glymphatic context requires direct quantification of solute transport or fluid dynamics. A series of recently developed non-invasive imaging techniques now permit these measurements in humans, notably CSF-STREAM [51] and 3D arterial spin labeling [52]. A caveat is that some of these methods rely on specialized infrastructure such as 7T MRI, and it may therefore be some time before they see broad clinical application. In the meantime, our findings position PVSV, FWI, and ALPS as robust correlates of sleep, age, and diurnal rhythm, suitable for further application in these domains when interpreted with due scientific caution. Given their reported relationship with a cavalcade of neuropathologies, these indices are likely best applied within large, integrative frameworks that combine demographic, neuropsychological, genetic, biomarker, and sleep measures to characterize the progression of complex disorders.

The present study had several limitations. First and foremost, the study's crux rested on the assumption that glymphatic exchange is reduced in both advanced age and sleep disturbance, a view that, while not universal, is supported by the majority of existing literature. Second, it featured a multi-site dataset with high variability across methodologies and outcomes. Although these differences were corrected for at the statistical level and with LOSO bootstrapping, some results remained sensitive to site-based variability. Third, the study was limited by several factors affecting the quantification and interpretation of the non-invasive MRI indices. For the ChPV, a relatively simple FreeSurfer-based segmentation approach was used, whereas more specialized segmentation methods may improve accuracy. For PVSV, segmentation procedures relied solely on T1w MRI, whereas including T2w MRI and FLAIR would have yielded better results. In addition, although the ShiVaI algorithm is trained to account for white matter hyperinstensities (WMH) and lacunes, we could not independently verify this correction. For the FWI, partial volume effects and WMH burden were not explicitly controlled for, and spatial normalization of FW maps to MNI space may have introduced interpolation-related biases. Finally, for the ALPS index, validity remains contested [25,26,42]; alterations in the microstructure or orientation of WM fibers remain a significant confounder; and the standardized ROIs applied here were not additionally validated by an external rater. While many of the above limitations were driven by data availability or the inherent constraints of the processing pipelines, future work should aim to address these limitations wherever possible. Fourth, due to lacking sufficient fMRI data, the study could not investigate functional non-invasive indices that have been proposed as proxies of glymphatic function, notably CSF-BOLD coupling [53]. Fifth, all MRI scans were obtained during wakefulness; future work could benefit from acquiring data during sleep to further elucidate associations with sleep. Sixth, the study did not incorporate objective measures of sleep quality, relying solely on subjective self-reports collected at the time of MRI acquisition. Future work could greatly benefit from methods such as polysomnography or actigraphy to improve the robustness of sleep-related findings. Seventh, the study combined acute and chronic sleep restriction into one condition, despite evidence that single-night and multi-night restriction paradigms exert diverging effects. This compromise was made to support balanced group comparisons with the sleep deprivation condition. To partially address this limitation, *post hoc* analyses comparing acute and chronic sleep restriction were conducted; however, the available data precluded a comprehensive examination, and further research with dedicated datasets will be necessary. Finally, the study employed a mixed-effects linear regression model with a normalized composite outcome to assess associated changes in a hypothetical latent system. This approach assumes equal weighting of all indices, whereas in reality individual alterations may vary in magnitude and timing. Future work should therefore consider more flexible modelling frameworks, such as latent variable models or dynamic factor models, to better capture potential heterogeneity in index alterations.

## Conclusion

Non-invasive MRI indices were altered under experimental sleep loss, age, and time of day, but sleep-related effects were dissociable from age-related alterations, suggesting that these measures capture distinct physiological processes rather than a shared underlying glymphatic mechanism.

## Methods

### Data Collection

The ENIGMA (Enhancing NeuroImaging Genetics through Meta-Analysis) consortium is a global initiative for data sharing in neuroimaging and genomics, encompassing over 400 institutions, 45 countries, and 50 specialized working groups [54]. The Sleep Working Group aims to address key questions in sleep research through collaborative data aggregation and analysis.

We compiled previously acquired MRI, demographic, cognitive, and sleep data of $n$ = 429 participants across 10 international sites [55–65]. All participants underwent two acquisitions of 3T T1w MRI and (optionally) dMRI: one after a night of habitual sleep and another following sleep restriction (SR) or sleep deprivation (SD). A small control sample ($n$ = 38) maintained normal sleep (NS) across both nights. Acquisition parameters are described in **Tables S2 and S3**. An overview of time of day differences between scanning sites is shown in **Figure S5**. Participants were excluded if they reported any chronic sleep disorders or any neurological or psychiatric disorders that typically affect sleep quality, or if they were under active treatment with neuropsychiatric or hypnotic medications.

### Sleep Restriction Protocol

The Christchurch [57], Cologne [56], Juelich [65], Stockholm [58], and Trondheim [63] datasets each employed a sleep restriction (SR) protocol (**Table S4**). After several days of naturalistic, habitual sleep with ca. 6 to 8 hours of time in bed (TIB), participants were restricted to a 3 to 6-hour TIB for up to five days. This was achieved by delaying sleep onset by ca. 3 hours to simulate real-life sleep deficits. MRI acquisition took place following one night of SR in the Christchurch, Singapore, and Stockholm datasets; three nights of SR in the Trondheim dataset; and five nights of SR in the Cologne and Juelich datasets.

### Sleep Deprivation Protocol

The Brussels [59], Cologne [55], Montreal [60], Oslo [61], Singapore [62], and Zurich [64] datasets employed a total sleep deprivation (SD) protocol (**Table S4**). Following several days of habitual sleep with a 6- to 8-hour TIB, participants were kept awake for 22 to 40 hours.

Participants typically engaged in quiet activities such as reading, watching non-arousing movies, or playing board games during their sleep deprivation period. Wakefulness was maintained by supervisory staff, with intermittent testing used in certain protocols to prevent dozing. In all studies, MRI acquisition occurred the day after one night of SD.

**Data Management**

Native DICOM files were converted into the BIDS format using dcm2bids version 3.2.0. BIDS verification was done using the containerized validator included in ReproNim. After verification, the raw data was stored as a DataLad dataset for easy management and version control. Dataset providence was recorded by DataLad and published on GitHub (https://github.com/jdelberse/enigma-sd).

**Anatomical Preprocessing**

All T1w images were preprocessed using *fMRIPrep* version 24.1.1. This fully automated pipeline is part of the *NiPreps* suite, which is continuously updated with the most recent best-practice preprocessing methods for MR images. The following summarizes the anatomical preprocessing steps performed by *fMRIPrep*: (1) bias field correction, to remove intensity inhomogeneities in the T1w image; (2) skull stripping, to remove non-brain tissue including the skull and surrounding anatomy; (3) tissue segmentation, to classify brain tissue into grey matter (GM), white matter (WM), and CSF; (4) spatial normalization, to warp the image into a standardized MNI template space; and (5) surface reconstruction, to estimate cortical surfaces, generate anatomical parcellations, and perform additional segmentations. By default, *fMRIPrep* generates a single, subject-level T1w reference image by merging across sessions. We instead employed a BIDS filter file to generate separate T1w images for the baseline and follow-up sessions.

**Diffusion Preprocessing**

All dMRI scans were preprocessed with *QSIPrep* version 0.24.1, a pipeline for diffusion-weighted data included in the *NiPreps* suite. The following summarizes the preprocessing steps performed by *QSIPrep*: (1) denoising, to reduce random noise and improve the signal-to-noise ratio; (2) unringing, to remove artifacts near tissue boundaries; (3) b=0 intensity harmonization, which normalizes intensities across b0 reference images; (4)

bias field correction; (5) susceptibility distortion correction; (6) eddy-current distortion correction, which realigns volumes to correct for head motion and eddy currents; (7) coregistration with T1w images; (8) spatial normalization; and (9) tissue segmentation.

**Quality Assurance**

As a final preprocessing step, all images underwent manual visual inspection. Participants were excluded in the event of visible artifacts or data corruption. Exclusions made during the preprocessing phase were made prior to the determination of the final subject counts reported in **Figure 1** and **Table 1**.

**MRI Indices**

***ChP Volume.*** The ChP volume (ChPV) and intracranial volume (ICV) were estimated by *FreeSurfer*'s automated segmentation module within the *fMRIPrep* workflow. The ChPV was subsequently divided by the ICV to account for intersubject variability in brain size.

***PVS Volume.*** The fully containerised *ShiVAi* pipeline was used to segment PVS in T1w images. This pipeline leverages the *SHIVA-PVS* model, a U-shaped neural network trained on the three-dimensional spatial features of PVS [66]. The pretrained weights for this model were obtained from the ShiVAi GitHub repository. The *FreeSurfer* Desikan-Killany parcellations were used to identify PVS clusters in the centrum semiovale (CSO), basal ganglia (BG), and hippocampus (HC). The total adjusted PVS volume (PVSV) was then calculated by summing individual PVS volumes and normalizing by the ICV to adjust for brain size. To assess segmentation accuracy, adjusted PVSVs were validated against volumes previously obtained by Custer and colleagues [31] in the Stockholm Sleepy Brains dataset [58].

***Free Water Index.*** The *Dipy* free water elimination model was used to obtain the free water-corrected fractional anisotropy and mean diffusivity, as well as a global free water estimate, from dMRI scans. Each of the scalar maps was then registered to standardised space using the native DWI-to-MNI transformation matrix obtained from *QSIPrep*. Finally, the free water index (FWI) was calculated by masking the global free water estimate with the WM segmentation obtained during anatomical preprocessing and averaging across the signal.

***DTI-ALPS.*** The DTI-ALPS (Diffusion Tensor Imaging Along the Perivascular Space) index was calculated as in Taoka et al. [23], where the MD in the x-direction along the projection and association fibres is divided by the MD in the y-direction (Dyy) and z-direction (Dzz) of the projection and association fibres, respectively. We used the ALPS script developed by Liu, Barisano, et al. [67] to perform this calculation on preprocessed dMRI data. Calculations were performed in MNI space using pre-defined regions of interest (ROIs) that were validated independently in prior work [67].

### Self-reported Sleep and Vigilance Measures

The Karolinska Sleepiness Scale (KSS) was administered across all datasets to assess daytime subjective sleepiness after SD or SR. It consists of a single 9-point item, ranging from 1 ("extremely alert") to 9 ("very sleepy, fighting sleep"). Additionally, the Psychomotor Vigilance Test (PVT) was administered to a subset (n = 202) during the sleep reduction protocol to assess sustained attention and vigilance. This test is a reaction-time task that measures the speed and variability of responses to visual stimuli presented at random interstimulus intervals. The present study utilized the mean reaction speed of participants, which was calculated by filtering out false starts (<100 ms) and lapses (>500 ms), taking the inverse of all remaining reaction times, and then calculating the average. Finally, the Pittsburgh Sleep Quality Index (PSQI) was administered to a subset (n = 181) to assess sleep quality over the last month. It consists of 19 items grouped into seven component scores: subjective sleep quality, sleep latency, sleep duration, habitual sleep efficiency, sleep disturbances, use of sleep medication, and daytime dysfunction. These components are summed to yield a total score between 0 and 21, with higher scores indicating poorer sleep quality.

Chronotype, that is, an individual's biologically driven preference for the timing of sleep and wakefulness within a 24-hour period, was determined using the Morningness-Eveningness Questionnaire (MEQ) or the Munich Chronotype Questionnaire (MCTQ). The MEQ consists of 19 items that evaluate sleep-wake preferences, alertness, and optimal times for physical and mental performance. The total score classifies individuals into one of five chronotypes, ranging from “definite morning type” to “definite evening type.”. These five categories were further reduced to three, yielding morning-types, evening-types, and intermediate-types. The MCTQ gathers detailed information on sleep-wake behavior, including mid-sleep time, social jetlag, and sleep debt. The central chronotype measure is mid-sleep on free days, corrected for sleep debt, with thresholds of <3:30 a.m. for morning-types and >5:30 a.m. for evening-types.

**Statistical Analyses**

To assess the impact of experimental SR and SDon MRI indices, data were restructured into a long format with three aligned vectors: sleep condition, MRI index category, and corresponding numerical index values. All index variables were z-normalized, and observations with $|z| > 3$ were excluded as outliers. DTI-ALPS values were sign-inverted to ensure that higher scores uniformly reflected greater abnormality across indices [30]. The z-normalized values were stacked into a single composite variable, with a categorical factor coding index identity, and analyzed with a linear mixed-effects (LME) model including sleep condition, index category, and their interaction as fixed effects. This approach models within- and between-subject variability, with the composite index reflecting coordinated changes across indices under the working assumption of a shared glymphatic system. Following systematic evaluation of potential covariates, age, sex, and time of day were included as fixed effects based on goodness-of-fit criteria. Scanning site was modeled as a random effect to account for substantial imbalances in dMRI availability across sites (**Table 1**) and site-related clustering of time-of-day values (**Figure S5**). To further mitigate potential site-related confounds, the main model was bootstrapped using a leave-one-site-out (LOSO) approach. A subject-level random intercept was included to account for repeated measures. To evaluate the performance of the main model and the composite index, four additional single-index LME models were fitted, using the z-normalized values of individual indices as the DV and the sleep condition as the IV. All models are outlined in **Table S1**.

The model-specific assumptions of linearity and homoscedasticity were assessed by plotting residuals against fitted values, and visual inspection indicated that these assumptions were satisfied. However, a QQ plot of residuals revealed deviations from normality in the main model, as confirmed by a Shapiro–Wilk test ($p < .001$). To address this violation, the main model was shifted to a robust linear mixed effects (RLME) framework. This method selectively downweights outlying observations to mitigate the influence of non-normal residuals and produce more stable estimates. Significance was evaluated using $t$-values, with $|t| > 1.96$ indicating a meaningful effect. Model coefficients (ß) were standardized post hoc to facilitate interpretation of effect sizes, with approximate $p$-values from the corresponding main LME model included for reference. Pairwise differences between sleep conditions were assessed for each index using estimated marginal means (EMMs) with confidence intervals (CIs).

**Resources**

BIDS conversion was performed using a custom virtual environment running Python 3.11.8. Data wrangling, analysis, and miscellaneous management tasks, e.g. the creation of unique subject identifiers, were carried out in a separate virtual environment running Python 3.11.11. MRI preprocessing workflows and DataLad operations were executed using Bash, and statistical analyses were performed in R 4.5.0. Figures were created using BioRender.com.

**Data Availability**

Data for this study were obtained from the ENIGMA-Sleep working group. The data are derived from previously published studies and are therefore subject to the data sharing policies of the original authors and institutions. Individual-level data are not publicly available due to ethical restrictions, including participants confidentiality and informed consent limitations. All analysis scripts and virtual environments have been made publicly available on GitHub (https://github.com/jdelberse/enigma-sd).

**Acknowledgements**

The authors would like to thank Gülru Horozoğlu for proofreading the manuscript and for providing guidance on the direction of this work.

**Funding**

This study did not receive any funding. J.D.E. is funded by the German Federal Ministry of Education and Research (BMBF) and the Max Planck Society. The Bruxelles sample was supported by the FNRS and the Fonds Wetenschappelijk Onderzoek – Vlaanderen (FWO) under the Excellence Of Science (EOS) Project (MEMODYN, No. 30446199 to P.P. and H.Z.). The Christichurch sample was supported by the Royal Society of New Zealand Marsden Fund (VDV0801), Canterbury Medical Research Foundation, Lottery Health Research(251144), and Accident Compensation Corporation (09/088), New Zealand. The Cologne samples were supported by the Institute for Scientific Information on Coffee (ISIC), the Swiss National Science Foundation, the Clinical Research Priority Program Sleep & Health of the University of Zurich, and the Aeronautics Program of the German Aerospace Center. The Juelich sample was supported by internal institutional funds and the project SleepLess, which received funding from the Bundesministerium für Bildung und Forschung (Federal Ministry of Education and Research, BMBF), the Fonds voor Wetenschappelijk Onderzoek - Research Foundation Flanders (FWO), and the Fonds de recherche du Québec - Santé (Quebec Health Research Fund, FRQS) under the frame of ERA-NET Neuron Cofund (grant # 01EW1808). The Montreal sample was supported by the Natural Sciences and Engineering Research Council of Canada and the Canada Foundation for Innovation. The MRI compatible high-density EEG device (Philips Neuro) and data acquisition were made possible through an internal grant from PERFORM center and the Faculty of Arts ans Science of Concordia University. The Oslo sample was supported by the Norwegian South-East Health Authorities (2018077, 2017090, 2015078), the Research Council of Norway (249795), the Centre for Digital Life Norway, the Ebbe Frøland foundation, the Norwegian Competence Center for Sleep Disorders, Haukeland University Hospital, Bergen (www.sovno.no), the University of Oslo Life Science summer scholarship for students and a research grant from Mrs. Throne-Holst. The Singapore sample was supported by the National Medical Research Council, Singapore (STaR/0004/2008), the National University of Singapore (NUS) Tier 1 Grant, the Singapore Ministry of Education Tier 2 Grant (MOE2014-T2-2-016), and the NUS Strategic Research Award. The Stockholm sample was supported by Riksbankens Jubileumsfond, Fredrik and Ingrid Thuring's Foundation, and the Karolinska Institutet Strategic Neuroscience Program. The Trondheim sample was supported by the Liaison

Committee between the Central Norway Regional Health Authority (RHA) and the Norwegian University of Science and Technology (NTNU) (2020/39645 to AO); and the National Institutes of Health (R01MH116147 to PMT). The Zurich sample was by the Swiss National Science Foundation grant #320030_163439 and the Clinical Research Priority Program Sleep & Health of the University of Zurich.

### Author Contributions

J.D.E. conceived and designed the study, collected the data from the contributing sites, developed the processing pipelines, performed the analyses, created the visualizations, and wrote the manuscript. D.Ap. assisted with study design and analyses. D.Ae., T.Å., A.B., F.B., K.B.B., C.C., N.C., S.G., C.G., S.C.H., J.L.O., H.S., S.T., N.Z., and W.S. contributed to data collection and carried out the original studies for each sample. P.P., M.C., T.T.D.-V., D.E., E.-M.E., T.E., C.v.G., H.-P.L., G.N., A.O., and G.P. were the principal investigators of the original studies. F.H. and L.W. assisted with data curation, including BIDS conversion, DataLad-based dataset management, and maintenance of online repositories. J.C. and R.C. assisted with the validation of PVS segmentations. A.Z.M., H.K., S.M.U.L., G.M.K., A.D., K.S., N.J., S.I.T., and P.M.T. are affiliated with ENIGMA and provided advice and facilitated data sharing. M.N. provided expert advice on the glymphatic system. J.J.I., S.B.E., and M.T. supervised the project. M.T. additionally contributed to the study design, established the ENIGMA-Sleep Working Group, and helped coordinate collaboration across sites. All authors critically revised the manuscript and approved the final version.

### Conflict of Interest

The authors declare no competing interests directly related to this work. S.C.H. is a full time employee of Hoffmann-La Roche. A.O. is a cofounder and share holder of Nordic Brain Tech AS.

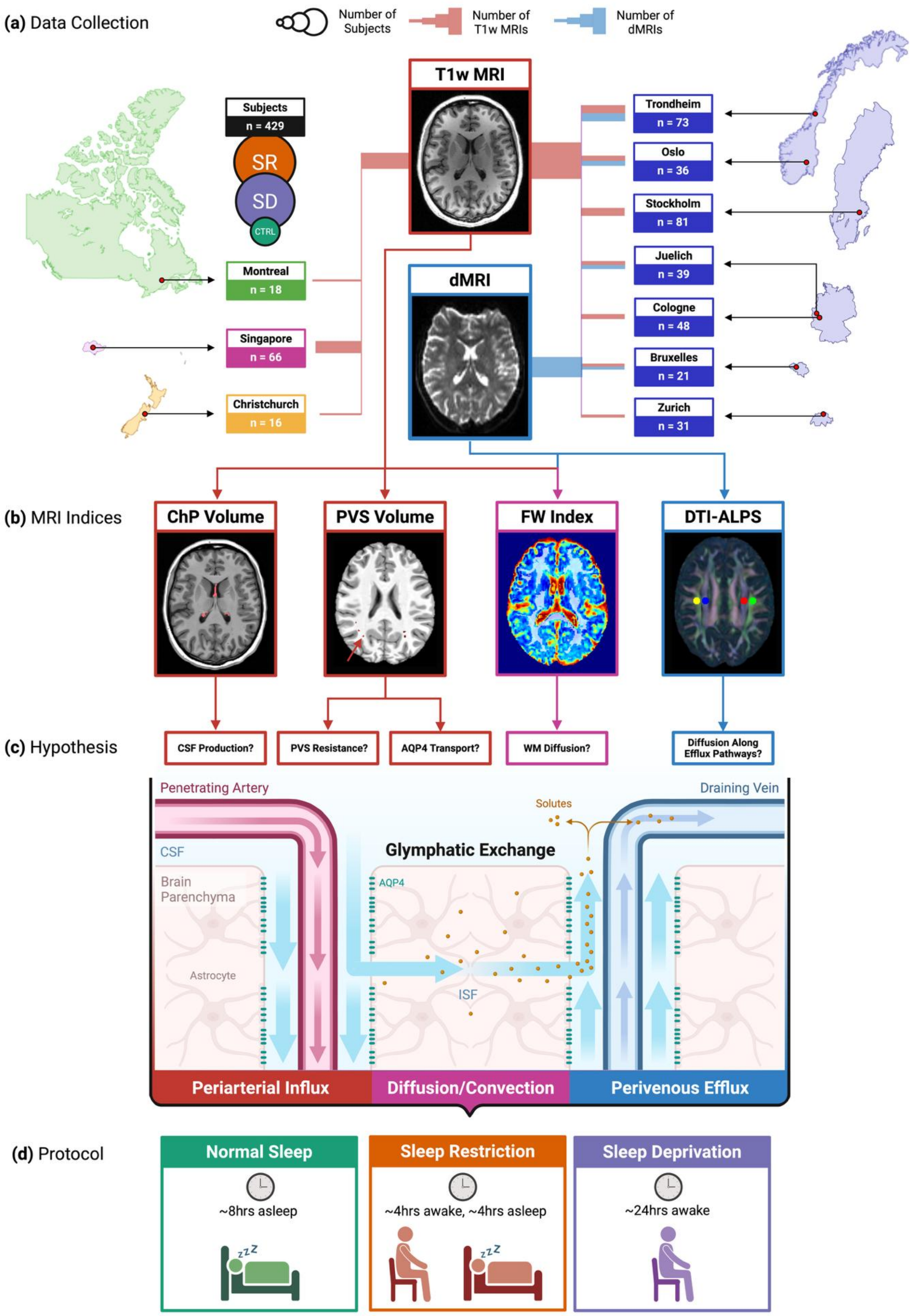

(a) Data Collection
Number of Subjects
Number of T1w MRIs
Number of dMRIs
T1w MRI
dMRI
Subjects
n = 429
SR
SD
CTRL
Montreal
n = 18
Singapore
n = 66
Christchurch
n = 16
Trondheim
n = 73
Oslo
n = 36
Stockholm
n = 81
Juelich
n = 39
Cologne
n = 48
Bruxelles
n = 21
Zurich
n = 31
(b) MRI Indices
ChP Volume
PVS Volume
FW Index
DTI-ALPS
(c) Hypothesis
CSF Production?
PVS Resistance?
AQP4 Transport?
WM Diffusion?
Diffusion Along Efflux Pathways?
Penetrating Artery
Draining Vein
Solutes
Glymphatic Exchange
CSF
Brain Parenchyma
AQP4
Astrocyte
ISF
Periarterial Influx
Diffusion/Convection
Perivenous Efflux
(d) Protocol
Normal Sleep
~8hrs asleep
Sleep Restriction
~4hrs awake, ~4hrs asleep
Sleep Deprivation
~24hrs awake

**Figure 1: Study overview.** (**a**) T1-weighted MRI and diffusion MRI data were acquired across ten international ENIGMA-Sleep sites. (**b**) These data were used to derive four non-invasive MRI indices: perivascular space volume, choroid plexus volume, the free water index, and the DTI-ALPS index. (**c**) Alterations in these indices have been proposed in prior studies to reflect different aspects of the glymphatic system, including periarterial CSF influx, parenchymal water diffusion, and perivenous efflux. (**d**) The present study investigates alterations in these MRI indices across three sleep conditions: normal sleep (ca. 8 hours of sleep), sleep restriction (ca. 4 hours of forced wakefulness followed by ca. 4 hours of sleep), and sleep deprivation (ca. 24 hours of forced wakefulness).

*ALPS = along the perivascular space, AQP4 = aquaporin-4, ChP = choroid plexus, CSF = cerebrospinal fluid, CTRL = control, dMRI = diffusion magnetic resonance imaging, DTI = diffusion tensor imaging, FW = free water, PVS = perivascular space, SD = sleep deprivation, SR = sleep restriction, T1w = T1-weighted, WM = white matter.*

| Dataset | Affiliation | Country | *N* | Age | %Male | Protocol | Images | Publications |
|---|---|---|---|---|---|---|---|---|
| **Bruxelles** | ULB Neurosciences Institute |  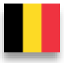 | 21 | 21.2 (2.5) | 47.6 | SD (22h) | T1w, dMRI | Stee et al.[59] |
| **Christchurch** | New Zealand Brain Research Institute |  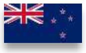 | 16 | 24.8 (5.1) | 50.0 | SR (3h TIB) | T1w | Poudel et al.[57] |
| **Cologne** | German Aerospace Center |  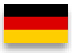 | 48 | 27.5 (4.9) | 60.4 | SD (24h)<br>SR (5h TIB) | T1w | Hennecke et al.[55]<br>Lin et al.[56] |
| **Juelich** | Forschungszentrum Juelich |  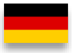 | 39 | 27.9 (6.2) | 33.3 | SD (24h) | T1w, dMRI | Elmenhorst et al.[65] |
| **Montreal** | Concordia University |  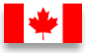 | 18 | 21.1 (2.7) | 38.9 | SD (36h) | T1w | Cross et al.[60] |
| **Oslo** | Oslo University Hospital |  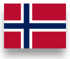 | 36 | 26.5 (6.9) | 38.9 | SD (32h) | T1w, dMRI | Voldsbekk et al.[61] |
| **Singapore** | National University of Singapore |  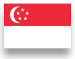 | 66 | 22.0 (2.3) | 50.0 | SD (24h) | T1w | Yeo et al.[62] |
| **Stockholm** | Karolinska Institutet |  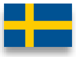 | 81 | 46.1 (22.6) | 49.4 | SR (3h TIB) | T1w | Nilsonne et al.[58] |
| **Trondheim** | Norwegian University of Science and Technology |  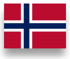 | 73 | 27.8 (5.3) | 47.9 | SR (-3h TIB) | T1w, dMRI | Smevik et al.[63] |
| **Zurich** | University of Zurich |  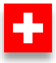 | 31 | 36.7 (19.0) | 100 | SD (40h) | T1w | Weigend et al.[64] |

**Table 1: Overview of participating ENIGMA-sleep sites**. Subject numbers are presented as total counts. Age is presented as means with standard deviation. Sleep deprivation protocols are defined by hours of forced wakefulness, while sleep restriction protocols are defined by hours spent in bed. *dMRI = diffusion magnetic resonance imaging, h = hours, SD = sleep deprivation, SR = sleep restriction, T1w = T1-weighted, TIB = time in bed.*

| | | Control | SR | SD | Total | *p* |
|---|---|---|---|---|---|---|
| **Total *N* (%)** | | 38 (8.9) | 205 (47.8) | 186 (43.4) | 429 | |
| **Age** | | 28.5 (7.4) | 34.2 (17.6) | 26.0 (10.0) | 30.1 (14.5) | <0.001*** |
| **Sex** | Female | 25 (65.8) | 105 (51.2) | 79 (42.5) | 209 (48.7) | 0.020** |
| | Male | 13 (34.2) | 100 (48.8) | 107 (57.5) | 220 (51.3) | |
| **Chronotype** | Morning | 6 (31.6) | 51 (25.0) | 20 (19.2) | 77 (23.5) | 0.012** |
| | Evening | 3 (15.8) | 42 (20.6) | 8 (7.7) | 53 (16.2) | |
| | Intermediate | 10 (52.6) | 111 (54.4) | 76 (73.1) | 197 (60.2) | |
| **PSQI score** | 0-5 | 11 (57.9) | 67 (75.3) | 49 (71.0) | 127 (71.8) | 0.453 |
| | 6-10 | 8 (42.1) | 21 (23.6) | 20 (29.0) | 49 (27.7) | |
| | 11-15 | 0 (0.0) | 1 (1.1) | 0 (0.0) | 1 (0.6) | |
| | 16-21 | 0 (0.0) | 0 (0.0) | 0 (0.0) | 0 (0.0) | |
| **BMI** | | 24.7 (2.8) | 23.6 (3.1) | 23.2 (2.9) | 23.5 (3.0) | 0.131 |
| **Handedness** | Left | 3 (15.8) | 5 (6.8) | 6 (15.8) | 14 (10.8) | 0.264 |
| | Right | 16 (84.2) | 68 (93.2) | 32 (84.2) | 116 (89.2) | |
| **Time of MRI Acquisition** | | 10:44 (1h20) | 14:13 (4h30) | 10:55 (2h47) | 12:28 (4h) | <0.001*** |

**Table 2: Participant demographics**. Continuous variables are presented as means with standard deviations. Categorical/ordinal variables are presented as counts with percentages. Time of day at MRI acquisition is reported in 24-hour clock format as HH:MM with standard deviations in hours and minutes. Comparisons between the control, sleep restriction, and sleep deprivation groups were conducted using analysis of variance (ANOVA) for continuous and chi-squared tests for categorical/ordinal variables. *ANOVA = Analysis of Variance, BMI = Body Mass Index, h = hours, MRI = magnetic resonance imaging, PSQI = Pittsburgh Sleep Quality Index, SD = Sleep Deprivation, SR = Sleep Restriction*

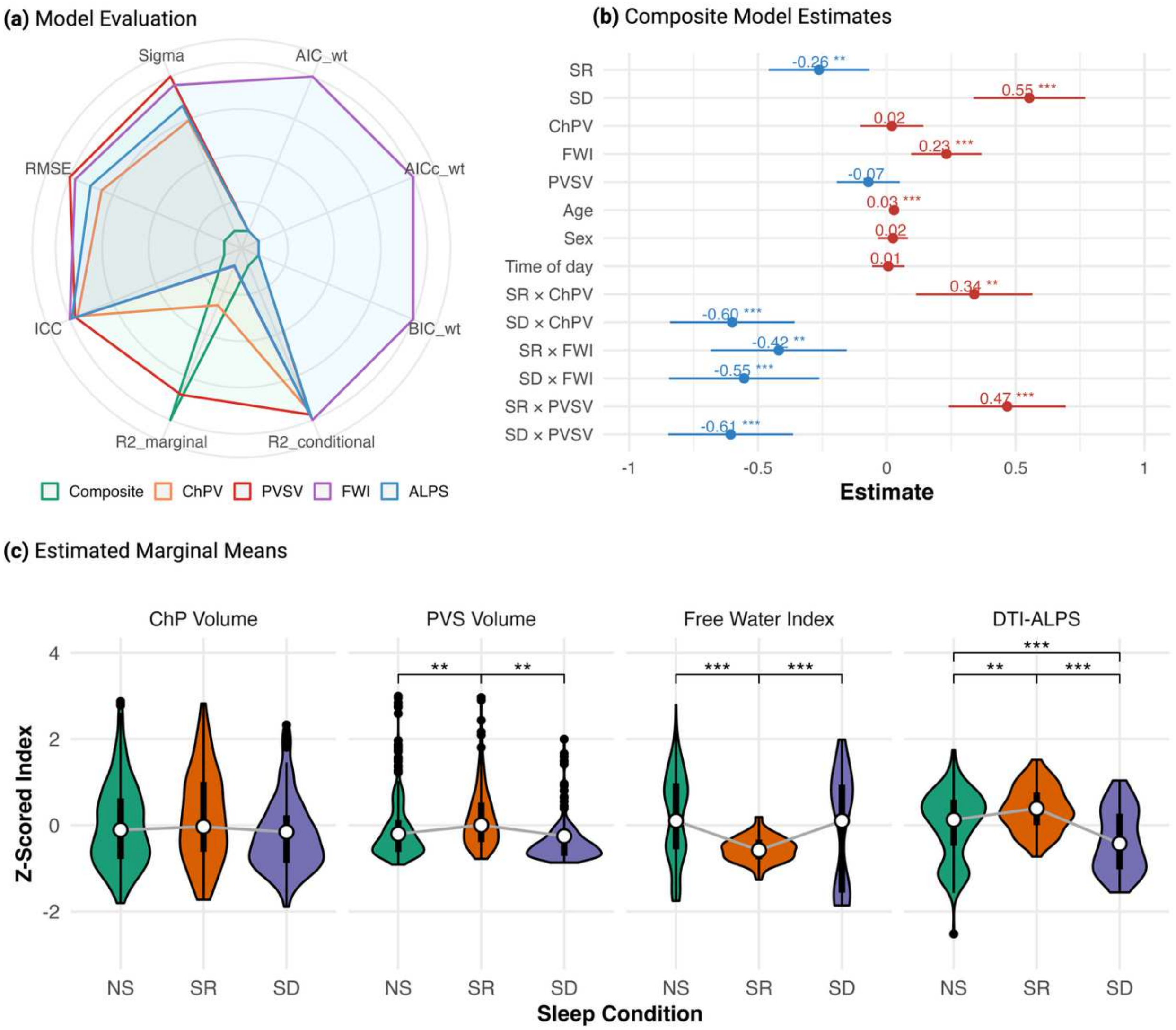

**Figure 2: Performance and outcomes of the composite index model.** (**a**) Evaluation of model performance for composite and single-index models. The composite index captured more variance through the fixed effects (higher marginal $R^2$), whereas information-criterion support favored the ALPS model and prediction accuracy favored all single-index models. (**b**) Fixed-effect estimates of the composite model. SR was associated with a significantly lower composite index, whereas SD and age were associated with a higher composite index. (**c**) Estimated marginal means from the composite model. SD was associated with a decrease in ALPS, whereas SR was associated with an increase in PVSV and ALPS, and a decrease in FWI. Statistical significance was defined as $p < 0.05$ following FDR correction.

*ALPS = along the perivascular space, AQP4 = aquaporin-4, ChP = choroid plexus, CSF = cerebrospinal fluid, CTRL = control, dMRI = diffusion magnetic resonance imaging, DTI = diffusion tensor imaging, FW = free water, PVS = perivascular space, SD = sleep deprivation, SR = sleep restriction, T1w = T1-weighted, WM = white matter.*

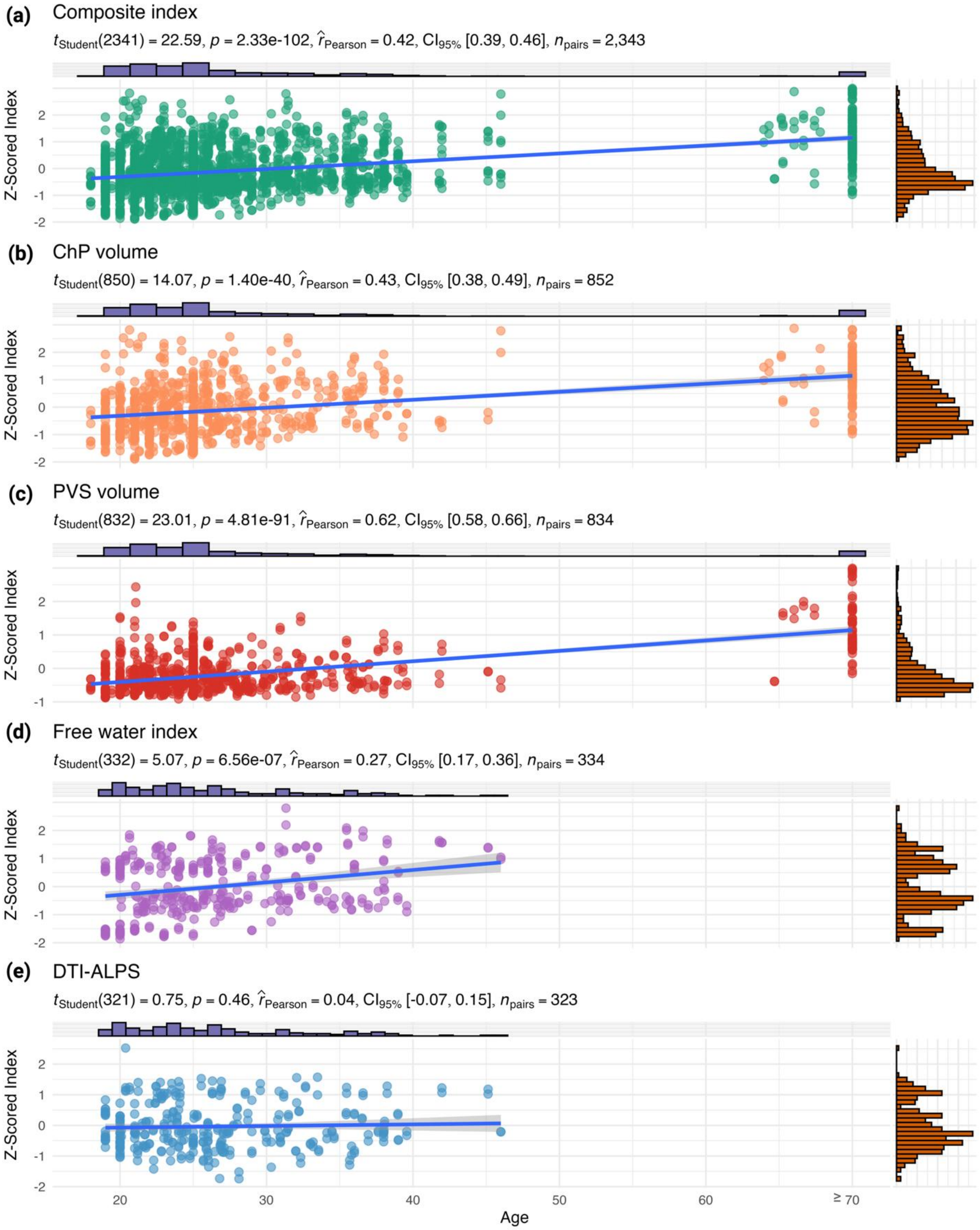

(a) Composite index
$t_{Student}(2341) = 22.59$, $p = 2.33e-102$, $\hat{r}_{Pearson} = 0.42$, $CI_{95\%}$ [0.39, 0.46], $n_{pairs} = 2,343$
(b) ChP volume
$t_{Student}(850) = 14.07$, $p = 1.40e-40$, $\hat{r}_{Pearson} = 0.43$, $CI_{95\%}$ [0.38, 0.49], $n_{pairs} = 852$
(c) PVS volume
$t_{Student}(832) = 23.01$, $p = 4.81e-91$, $\hat{r}_{Pearson} = 0.62$, $CI_{95\%}$ [0.58, 0.66], $n_{pairs} = 834$
(d) Free water index
$t_{Student}(332) = 5.07$, $p = 6.56e-07$, $\hat{r}_{Pearson} = 0.27$, $CI_{95\%}$ [0.17, 0.36], $n_{pairs} = 334$
(e) DTI-ALPS
$t_{Student}(321) = 0.75$, $p = 0.46$, $\hat{r}_{Pearson} = 0.04$, $CI_{95\%}$ [-0.07, 0.15], $n_{pairs} = 323$
Z-Scored Index
Age
20
30
40
50
60
≥ 70

**Figure 3: Correlations between age and MRI indices,** including the (**a**) composite index, (**b**) choroid plexus volume, (**c**) perivascular space volume, (**d**) free water index, and (**e**) DTI-ALPS. Side histograms show the distribution of values. All indices except DTI-ALPS increased significantly with age. Ages ≥70 were top-coded prior to the present study. Significance was assessed using a Student's t-test of the Pearson correlation and defined as $p < 0.05$ following FDR correction.

*ALPS = along the perivascular space, AQP4 = aquaporin-4, ChP = choroid plexus, CSF = cerebrospinal fluid, CTRL = control, dMRI = diffusion magnetic resonance imaging, DTI = diffusion tensor imaging, FW = free water, PVS = perivascular space, SD = sleep deprivation, SR = sleep restriction, T1w = T1-weighted, WM = white matter.*

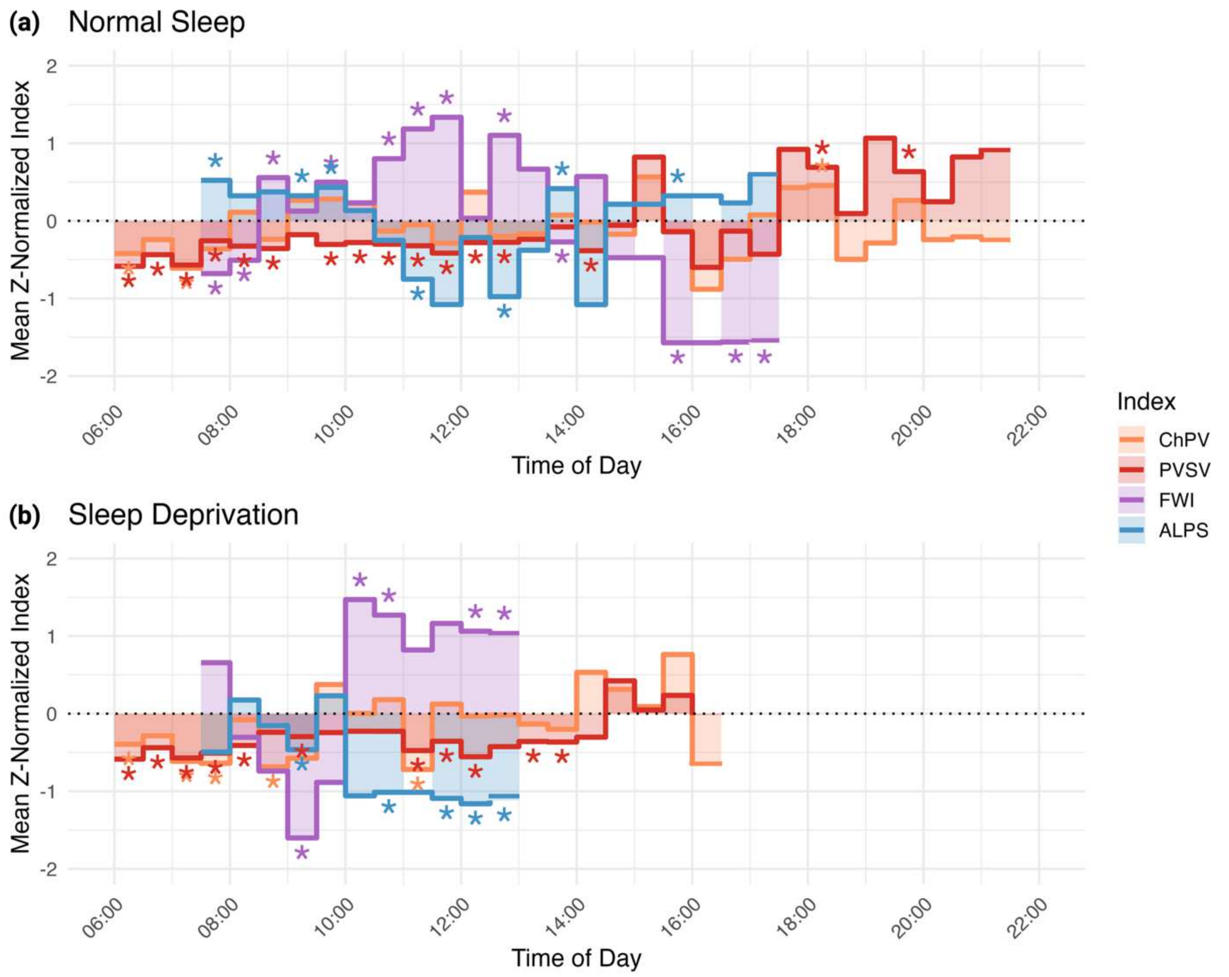

**Figure 4: Diurnal changes of MRI indices** following (a) normal sleep and (b) sleep deprivation. Normalized MRI index values were aggregated in 30-minute time bins. Statistical significance at each time bin was assessed using one-sample t-tests against zero, with bins containing fewer than two data points excluded. Diffusion measures were elevated in the early morning and afternoon and attenuated in the late morning. PVS volumes were lowest in the morning and peaked in the late evening. ChP volumes remained relatively stable throughout the day. Interestingly, ALPS and FWI appeared temporally anticorrelated. Statistical significance was defined as $p < 0.05$ following FDR correction.

*ALPS = along the perivascular space, ChP(V) = choroid plexus (volume), DTI = diffusion tensor imaging, FDR = false discovery rate, FWI = free water index, PVS(V) = perivascular space (volume)*

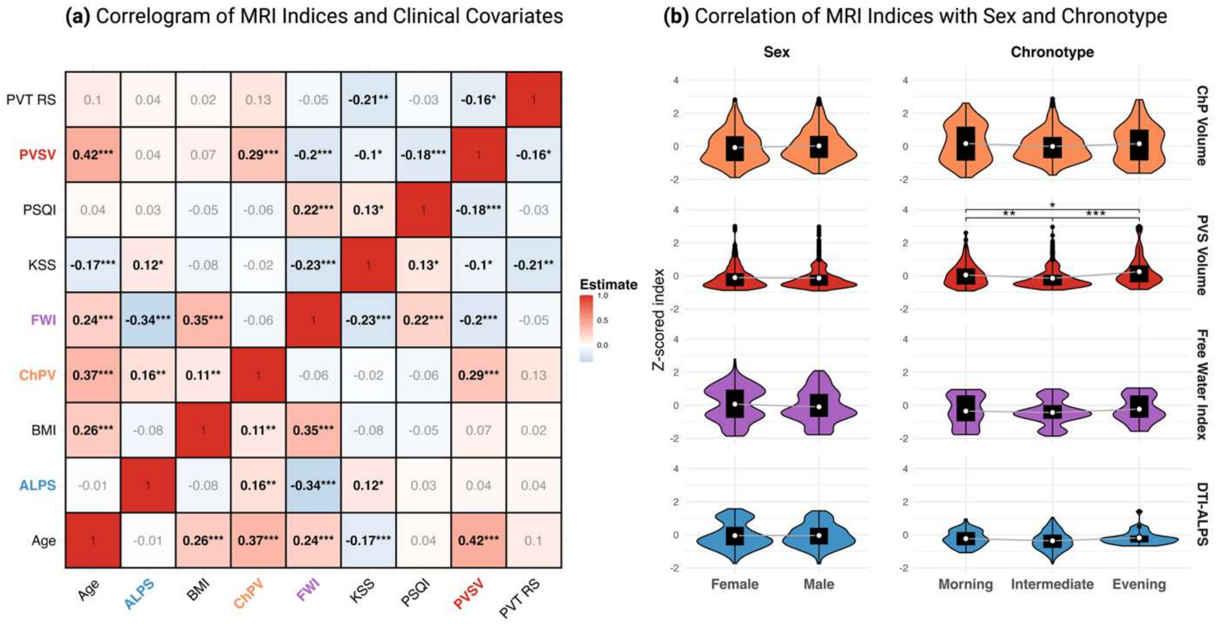

**Figure 5: Post hoc correlations.** (**a**) Spearman correlation matrix showing associations between demographic variables (age, BMI), sleep and cognitive measures (PSQI, KSS, PVT reaction time), and MRI indices (ChPV, PVSV, FWI, ALPS). (**b**) Group differences in sex and chronotype by MRI indices, assessed using independent-samples t-tests and pairwise t-tests, respectively. Statistical significance was defined as $p < 0.05$ following FDR correction.

*ALPS = along the perivascular space, BMI = body mass index, ChP(V) = choroid plexus (volume, FDR = false discovery rate, FWI = free water index, KSS = Karolinska sleepiness scale, PSQI = Pittsburgh sleep quality index, PVS(V) = perivascular space (volume), PVT = psychomotor vigilance task, RS = reaction speed.*

## Supplementary Files

This is a list of supplementary files associated with this preprint. Click to download.

- ENIGMASDmanuscriptsupplement.pdf
